# 3D geometric reconstruction of thoracic aortic aneurysms

**DOI:** 10.1186/1475-925X-5-59

**Published:** 2006-11-02

**Authors:** Alessandro Borghi, Nigel B Wood, Raad H Mohiaddin, X Yun Xu

**Affiliations:** 1 Department of Chemical Engineering, South Kensington Campus, Imperial College London, UK; 2 Royal Brompton and Harefield NHS Trust, Sydney Street, London, UK

## Abstract

**Background:**

The thoracic aortic aneurysm (TAA) is a pathology that involves an expansion of the aortic diameter in the thoracic aorta, leading to risk of rupture. Recent studies have suggested that internal wall stress, which is affected by TAA geometry and the presence or absence of thrombus, is a more reliable predictor of rupture than the maximum diameter, the current clinical criterion. Accurate reconstruction of TAA geometry is a crucial step in patient-specific stress calculations.

**Methods:**

In this work, a novel methodology was developed, which combines data from several sets of magnetic resonance (MR) images with different levels of detail and different resolutions. Two sets of images were employed to create the final model, which has the highest level of detail for each component of the aneurysm (lumen, thrombus, and wall). A reference model was built by using a single set of images for comparison. This approach was applied to two patient-specific TAAs in the descending thoracic aorta.

**Results:**

The results of finite element simulations showed differences in stress pattern between the coarse and fine models: higher stress values were found with the coarse model and the differences in predicted maximum wall stress were 30% for patient A and 11% for patient B.

**Conclusion:**

This paper presents a new approach to the reconstruction of an aneurysm model based on the use of several sets of MR images. This enables more accurate representation of not only the lumen but also the wall surface of a TAA taking account of intraluminal thrombus.

## Background

An aortic aneurysm is an abnormal enlargement of a portion of the aorta, due to the progressive weakening of the aortic wall. TAAs, aneurysms involving the aorta in the thoracic area, are characterized by low frequency (0.006% in a given population) but very high mortality rate (39–62% of the diagnosed cases [1]). The only criterion for the selection of surgical patients is based on the maximum diameter of the aneurysm, which has been proved not to be completely reliable for the assessment of the rupture risk. Furthermore, recent studies focused on abdominal aortic aneurysms have shown that peak wall stress in the aortic wall, calculated by means of the finite element method, is a more reliable parameter [2].

The latest trend of the research in this field is to make use of image-based geometries in order to calculate patient-specific wall stress patterns [3-6]. The segmentation of the aortic lumen and wall is a crucial step for the creation of a finite element model. The reconstruction of arterial structures involves the use of sets of clinical data (MRI or CT, in general) that are processed to extract the vessel morphology. While the segmentation of the arterial lumen is a well established technique and has been performed with different modalities in living subjects [7-10], the segmentation of the wall and its connective components is still a challenge due to the low contrast between the wall and the surrounding tissues [11]. The extraction of wall information has been attempted for the carotid and coronary arteries, using ex vivo imaging data [12-15]. As pointed out in these studies, the difficulty of achieving equivalent results with in vivo data is the frequent inability to acquire arterial wall images with a sufficient resolution [15]. Good results were achieved by Thomas et al. [16], who applied the discrete dynamic contour algorithm to four subjects scanned at the left carotid artery using the black blood MRI technique. The results showed that while the SNR (signal to noise ratio) improved with the increase of the field of view (FOV), the SDNR (signal difference to noise ratio, a measure of the capability of a MR protocol to generate contrast between different tissues) is independent of the dimension of the FOV. Further work has been done using semi-automatic techniques applied to the segmentation of arterial structures, with good results for abdominal aortic aneurysms [11,17,18]. However, these methods are based on the use of CT images, where good differentiation between the thrombus and wall may be possible should there be calcifications inside the wall. Furthermore, all the studies on the application of these methods to the segmentation of thrombus show, with good agreement, that automatically segmented contours do not always match manual segmentation performed by experienced operators, and reducing the degree of user intervention does not necessarily correspond to a higher accuracy in contour depiction and volume measurement [11].

This paper presents a new approach to the reconstruction of patient-specific aneurysm models, based on the use of different MR imaging sequences. Two sets of images, with different specifics and resolutions, were used in order to create a model with the best level of anatomical details for both the wall and lumen. The 3D aneurysm model was then reconstructed using a CAD program and subsequently imported into a commercial finite element software for mesh generation and stress analysis. This method has been applied to two aneurysm models and the results have been compared with the less accurate models constructed from a single set of images.

## Methods

Two patients with TAAs were involved in this study. Both patients have aneurysms in the descending thoracic aorta, superior to the diaphragm. According to the Crawford classification [19], these patients have type I thoracoabdominal aneurysm (the aneurysm originates below the subclavian artery and involves most of the thoracic aorta). The patients were scanned for TAA examination, at the Royal Brompton Hospital, London, using a Siemens 1.5T MR scanner. The study conformed to the Declaration of Helsinki; although ethical approval for the project was obtained from the local ethics committee, the scans performed on the patients were requested on clinical grounds. Multiple 2D HASTE (half-Fourier acquisition single-shot turbo spin-echo) images and 3D contrast enhanced MR angiograms (CE-MRA Sequence, with spoiled gradient echo sequence) were acquired for both patients, in order to assess site and shape of the aneurysms. HASTE images are suitable for the delineation of arterial wall and its composition (see [20,21]). The acquisition of these images was cardiac gated and the set of images corresponding to a diastolic phase was used; for this reason it was taken as the reference configuration in this study. The inter-slice distance for the first patient was double that of the second patient (see Table 1), since Patient A had a much longer aneurysm. The images of the sets under consideration (A1 and B1) were acquired on clinical grounds and were taken such that the scan time was kept as short as acceptable. Thus, only a limited number of images could be acquired.

**Table 1:** Image summary. Summary of the images used for model creation

	PATIENT A		PATIENT B	
	Set A1	Set A2	Set B1	Set B2

Image type	HASTE	CE MRA	HASTE	CE MRA

Slice thickness (mm)	6	1.5	6	1.5
Number of slices	20	200	30	200
TR (ms)	800	2.84	660	2.84
TE (ms)	23	1.04	23	1.04
Pixel resolution (mm)	1.38	0.69	1.38	0.69
Slice distance (mm)	12	0.8	6	0.8

For the acquisition of the second type of images contrast agent was injected intravenously in both subjects; oblique – sagittal planes encompassing the entire thoracic aorta were acquired. Maximum intensity projections and multi-planar reconstructions were performed on the acquired data. This technique eliminates the signal of the static tissues by means of a double sequence of acquisition before and after the injection of contrast agent [22]. Table 1 summarizes the information about the patients and the images used for model reconstruction.

**Figure 1:**
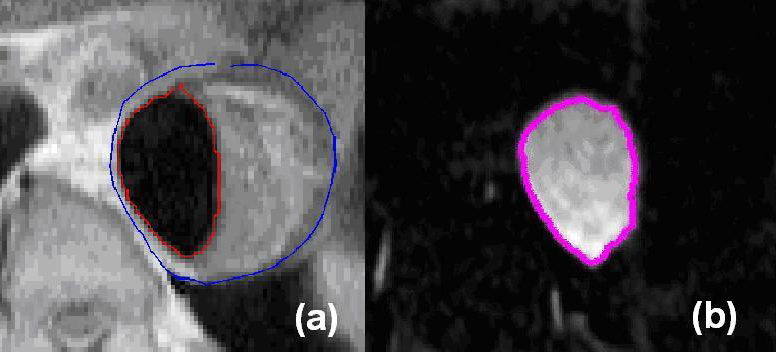
**Image segmentation samples**. Examples of the MR images and segmented contours of Set A1 (figure 1a) and Set A2 (figure 1b) for patient A.

The procedure for model creation is described and illustrated here using images acquired from patient A, but the same approach has been applied to data for patient B. The first step consisted of delineating the lumen and outer wall of the aneurysm from image set A1. This was performed by a manual segmentation procedure implemented via a MATLAB program, yielding contours of the lumen (c_LH_) and outer wall (c_W_). Image set A2 was segmented using a semi-automatic approach based on the region growing method, due to good contrast between the lumen and its surroundings. The results of segmentation of set A2 were lumen contours c_LM_. Figure 1 shows a pair of sample images from sets A1 and A2 as well as their segmentation results.

**Figure 2:**
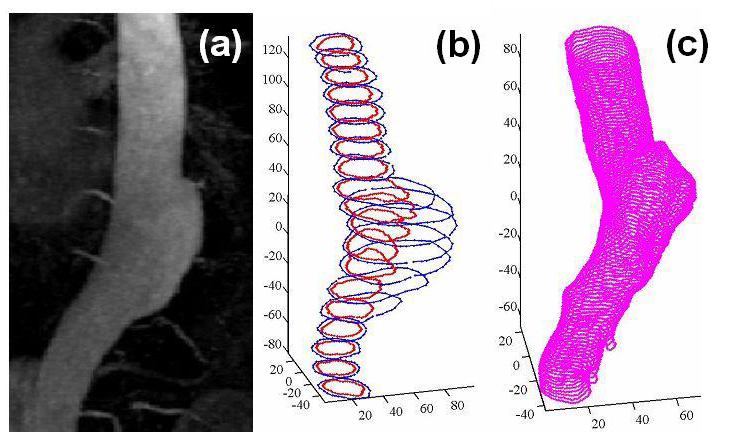
**Segmentation results**. From left to right: (a) 3D MR angiogram of the TAA of Patient A; (b) aneurysm shape from the segmentation of the image set A1 (wall contours c_W _in blue and lumen contours c_LH _in red); (c) lumen of the aneurysm from the segmentation of image set A2 (lumen contours c_LM _in magenta) (Units in mm).

Figure 2 shows respectively a 3D MR angiogram of the aneurysm of patient A, stacked contours c_W _and c_LH_, and stacked contours c_LM_. As can be noticed, the 3D shape of the lumen formed by the c_LM _contours resembles more closely the real geometry (shown in Figure 2a) than the one defined by c_LH_, due to higher spatial resolution with contrast-enhanced MRA.

**Figure 3:**
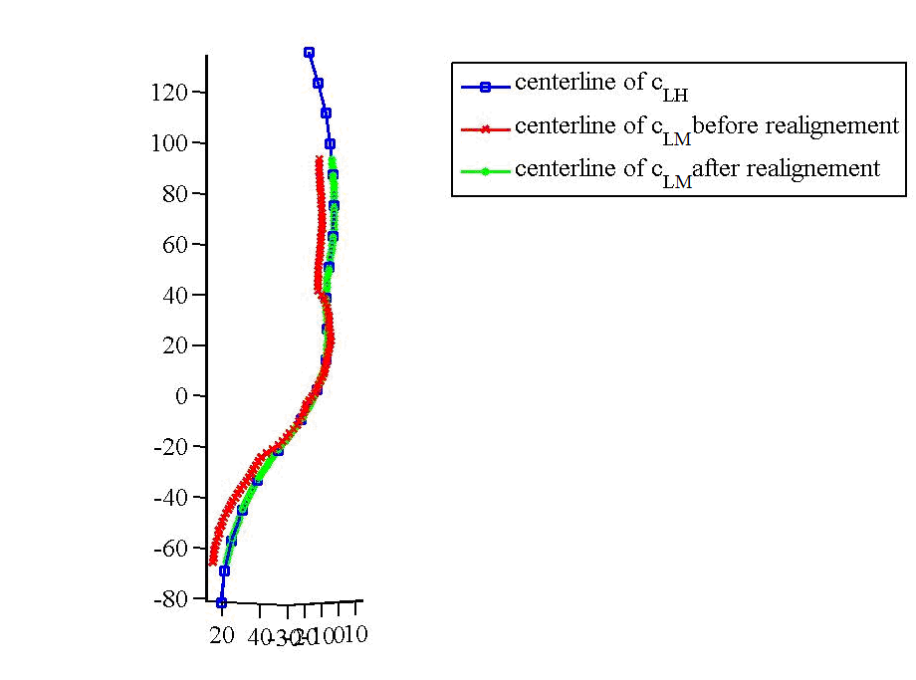
**centreline realignment**. Realignment of the centreline (Patient A) (Units in mm).

**Figure 4:**
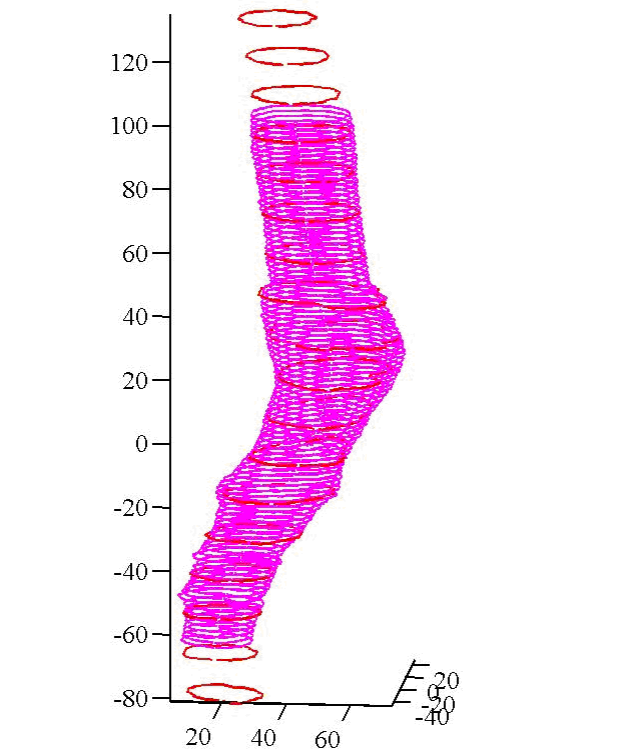
**Lumen shape comparison**. Comparison of the lumen shape from set A1 (contour c_LH_, in red) and set A2 (contour c_LM_, in magenta) (Patient A) (Units in mm).

After the segmentation was completed, two aneurysm models were created: model H formed by the lumen and wall contours extracted from set A1 (HASTE images: 'H'), and model HM defined by the lumen contours from set A2 (CE-MRA: 'M') combined with the wall contours from A1 ('H'). Since images A1 and A2 were not acquired at the same time, registration of the corresponding slices was necessary before building model HM. This was performed by co-locating the centre points of contours c_LM _with their counterparts in contours c_LH_, after centreline smoothing of c_LH_. Figure 3 shows the comparison of the centrelines of c_LH _and c_LM _before and after centreline registration. This was followed by translating contours c_LM _to match their new centre points. Figure 4 shows the comparison and matching of the lumen contours c_LH _and c_LM _after translation.

For the creation of the CAD models, the number of slices contained in c_LM _was reduced from 200 to 50 slices, while c_LH _and c_W _were interpolated from 20 to 50 contours in order to have the same vertical coordinates as c_LM_.

**Figure 5:**
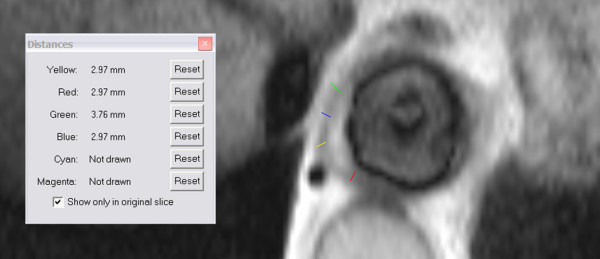
**Wall thickness retrieval**. Example of the wall thickness measurement (Patient B).

Due to the low contrast between arterial wall and intra-luminal thrombus, it was not possible to distinguish the wall from thrombus for every slice, so the wall was assumed to have a constant thickness along the entire aneurysm. Therefore, the wall thickness was determined by taking the average value of 12 measurements made at sections where wall thickness was clearly defined (usually in the region without thrombus), similar to the approach adopted by Wang et al. [23]. The resulting wall thickness was 3.44 ± 0.42 mm for patient A and 3.05 ± 0.35 mm for patient B. Figure 5 shows an example of the wall thickness measurement.

Using the wall thickness determined by the above described procedure, the inner wall contours (c_T_, representing the boundary between the aortic wall and thrombus) were created by shrinking the outer wall contour (c_W_) uniformly to match the measured wall thickness. Local adjustment might be needed in regions where the radial distance between the wall and lumen contours was smaller than the measured average wall thickness. This was achieved by introducing a special function which checks the radial distance between the newly formed inner wall and lumen contours against a specified threshold. If the distance is lower than the threshold value, the point on contour c_T _will be moved outwards by a set distance. The detailed procedure can be summarized as follows:

- transformation of inner wall and lumen contours from Cartesian (x_L_-y_L _for the lumen and x_T_-y_T _inner wall) to polar coordinates (ρ_L_-θ_L _for the lumen and ρ_T_-θ_T _for the thrombus);

- identification of the points of the lumen that are contained in the angular portion defined by

|*θ*_*T *_- *θ*_*L*_| <*a*

- identification of the points that fail the minimum distance test

*ρ*_*T *_- *ρ*_*L *_<*b*

- readjustment of the radial coordinate ρ_T _of these points by adding to their original distance an adjustment distance *c*

*ρ'*_*T *_= *ρ*_*T *_+ |*ρ*_*T *_- *ρ*_*L*_| + *c*

- transformation of the newly calculated points from polar coordinates (ρ'_T_-θ_T _for the readjusted inner wall) to Cartesian coordinates (x'_T_-y'_T_).

**Figure 6:**
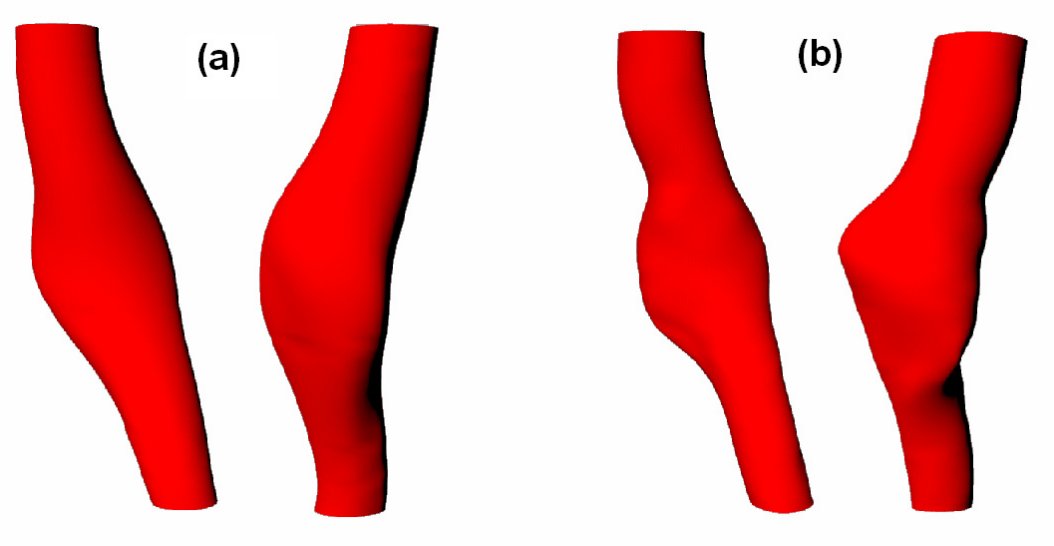
**Lumen surface for the two different models**. Comparison of the lumen surface from set A1 (figure 6a) and set A2 (figure 6b) for Patient A.

**Figure 7:**
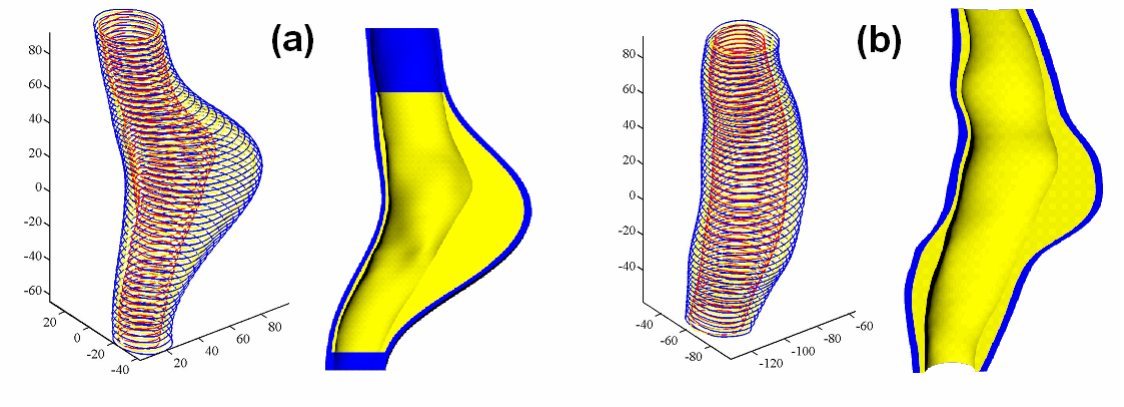
**CAD models**. TAA models for Patient A (figure 7a) and Patient B (figure 7b). The contours are from the MATLAB segmentation, and the surface rendering are from the CAD models, with the wall domain shown in blue and the thrombus domain shown in yellow (Units in mm).

The parameters a, b and c were chosen in order to avoid overlapping between the final inner wall contour and the lumen after smoothing. The values for both patients were a = 90 degrees, b = 0.5 mm, c = 0.8 mm. The sensitivity of the numerical solution to these parameters was assessed and will be discussed later. As a result, two additional sets of contours were obtained: c_TH_, the boundary between the thrombus and wall with lumen contours given by c_LH_, and c_TM_, with lumen contours defined by c_LM_. The contour points were imported into a CAD program (Rhinoceros, 2003 Robert McNeel & Assoc.) to create the 3D solid model. The H model was created by using contours c_W_, c_TH _and c_LH_, while the HM model used contours c_W_, c_TM _and c_LM_. Comparison of the H and HM models is given in Figure 6, which shows that the H model failed to capture some fine geometric features, due to larger slice thickness and slice distance. Figure 7 shows the geometrical reconstruction of the aneurysms for patients A and B created with contours c_W_, c_TM _and c_LM _along with the CAD models.

The CAD models were imported into ADINA 8.2 (ADINA R&H Inc.), a validated finite element package suitable for fluid-solid interaction simulations. Four models were built and details of these are summarised in Table 2. The thrombus and wall were subdivided using tetrahedral elements. The material properties were retrieved from the literature and both the wall and thrombus were modelled as a homogeneous hyperelastic material with a stress-stretch relationship given by

**Table 2:** Model creation summary. Summary of the contours used for the creation of the four models.

*Model*	*Patient*	*Lumen contour*	*Thrombus contour*	*Wall Contour*
A_H_	A	c_LH_	c_TH_	c_W_
A_HM_	A	c_LM_	c_TM_	c_W_
B_H_	B	c_LH_	c_TH_	c_W_
B_HM_	B	c_LM_	c_TM_	c_W_

*T *= [2*α*_*W *_+ 4*β*_*W*_(*λ*^2 ^+ 2*λ*^-1 ^- 3)]·(*λ*^2 ^- *λ*^-1^)

for the aortic aneurysm wall [24] and

*T *= [2*α*_*T *_+ 4*β*_*T*_(2*λ *+ *λ*^-2 ^- 3)]·(*λ *- *λ*^-2^)

for the thrombus [25], where T is the stress and λ is the stretch ratio. For ascending TAA strips, Vorp et al. [26] found values for the constants as α_W _= 100 kPa and β_W _= 530 kPa while for intra-luminal thrombus Wang et al [25] found α_T _= 28 kPa and β_T _= 28.8 kPa.

Mesh generation for the thrombus and wall domains were carried out separately so it was necessary to define the interface as 'tied contact boundary' in order to hinder sliding and independent movement. Both ends of the models were constrained to avoid rigid translation.

A grid independence test was performed for each model and a maximum difference of 5% in displacement and stress between the adopted and a finer mesh was accepted.

## Results

**Figure 8:**
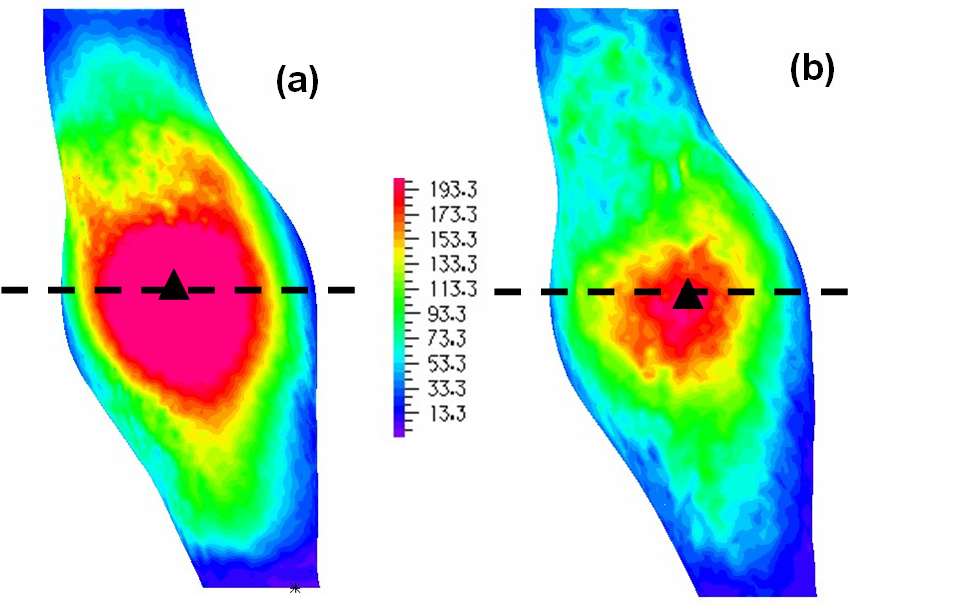
**Patient A, stress pattern on the outer wall**. Comparison of wall stress patterns on the outer wall for Patient A (A_H _in figure 8a, A_HM _in figure 8b); the dotted lines show the sections where transverse stress distributions are resented in figure 9, the triangles show the points of maximum stress (Units in kPa).

**Figure 9:**
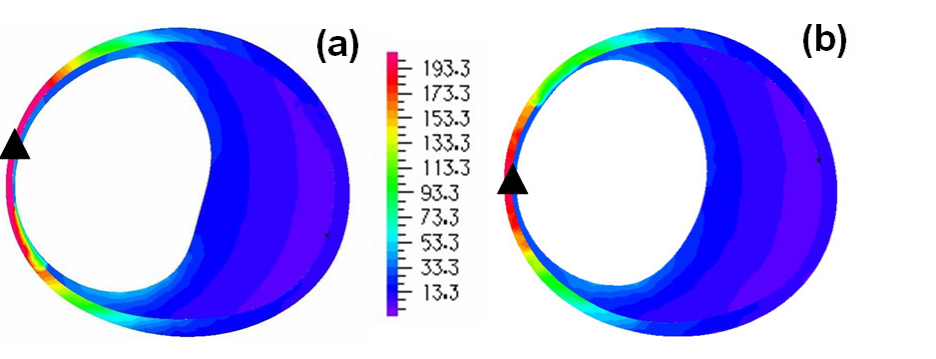
**Patient A, stress pattern on a horizontal section**. Comparison of cross-sectional wall stress distributions at the locations of maximum stress for Patient A (A_H _in figure 9a, A_HM _in figure 9b); the triangles show the point of maximum stress (Units in kPa).

Four TAA models, two for each patient, were generated and analysed here (see Table 2 for model details). As explained earlier, the two models for each patient have the same outer wall surface but differ in lumen surface details and distribution of intraluminal thrombus. The effects of these geometrical differences on predicted stress patterns were examined by performing solid stress analysis of these models under the same loading and boundary conditions. A static uniform internal pressure of 120 mmHg (representing normal systolic blood pressure) was applied. Figure 8 shows the predicted stress patterns on the anterior walls of models A_H _and A_HM _(for patient A). Similar stress patterns were found but model A_H _gave a higher stress level (by 30%) than model A_HM_. This was supported by stress distributions in the transverse sections at locations where maximum stress was found (Figure 9, with locations of the sections indicated in Figure 8). The region of high stress in model A_H _is larger than in A_HM _because of the different thrombus distribution given by the different lumen details determined for two models. Stress patterns within the thrombus layer are similar since the stress level there is relatively low, primarily due to increased wall thickness in this area and secondarily to the high thickness of the thrombus layer.

**Figure 10:**
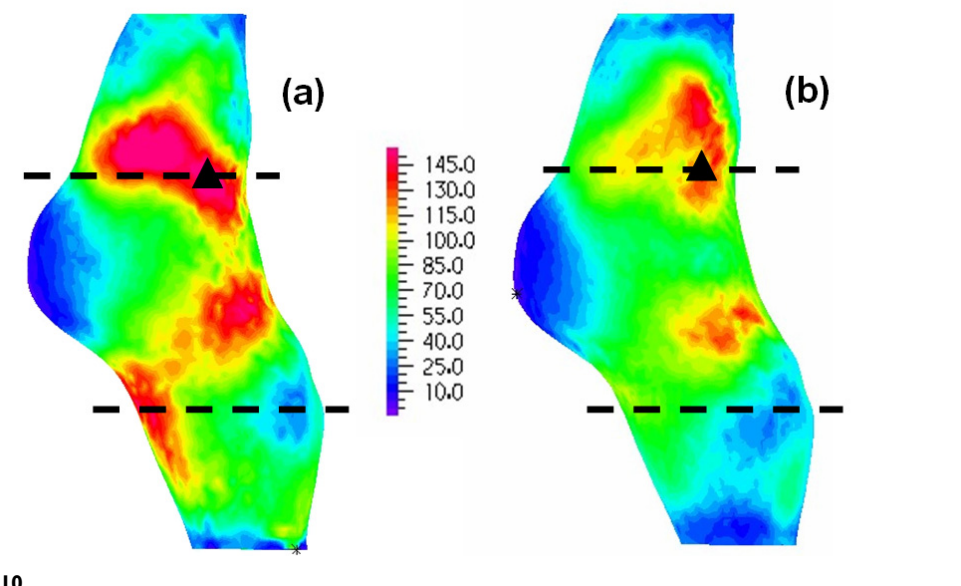
**Patient B, stress pattern on the outer wall**. Comparison of wall stress patterns on the outer wall for Patient B (B_H _in figure 10a, B_HM _in figure 10b); the dotted lines show the sections where transverse stress distributions are presented in figure 11, the triangles show the points of maximum stress (Units in kPa).

**Figure 11:**
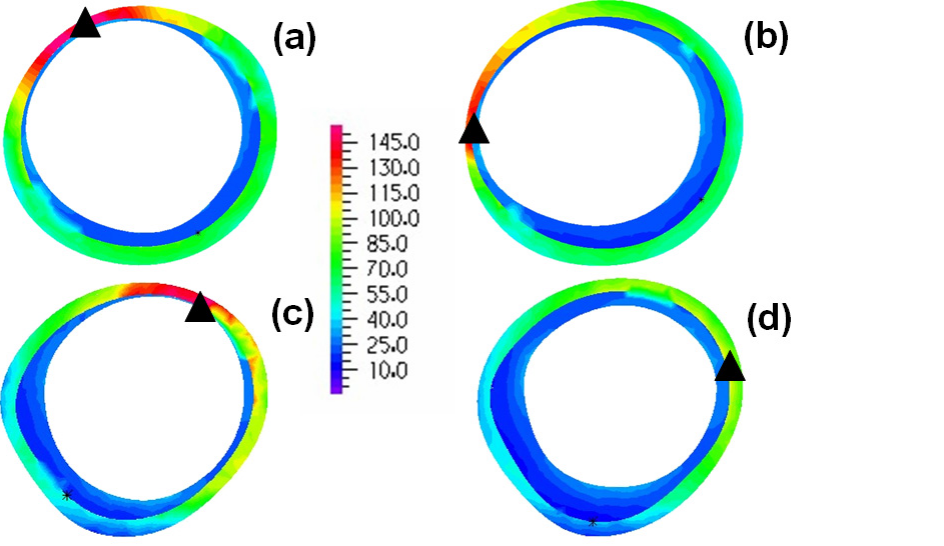
**Patient B, stress pattern on horizontal sections**. Comparison of cross-sectional wall stress distributions at two locations for Patient B; sections from model B_H _are given on the in figure 11a and 11c and sections from model B_HM _are in figure 11b and 11d. The triangles show the points of maximum stress at the sections (Units in kPa).

Figures 10 and 11 show the predicted stress patterns for models B_H _and B_HM _(patient B) on the posterior wall and transverse sections respectively. Similar to observations made with models for patient A, the stress patterns agree qualitatively but quantitative differences exist, with higher stress values in model B_H _(the maximum stress in the B_H _model is 11% higher than in the B_HM _model). Figure 11 gives the comparison of stress distributions at two sections along the aneurysm, as indicated in figure 10 with dotted lines. Stress patterns in these models are strongly dependent on the distribution of thrombus and lumen shape. In the upper section the high stress regions differ slightly and in both models the maximum stress is located where the thrombus is the thinnest. In the lower section, areas of high stress are present in model B_H _but are absent in model B_HM_, due to the different shape of intraluminal thrombus at this section.

**Figure 12:**
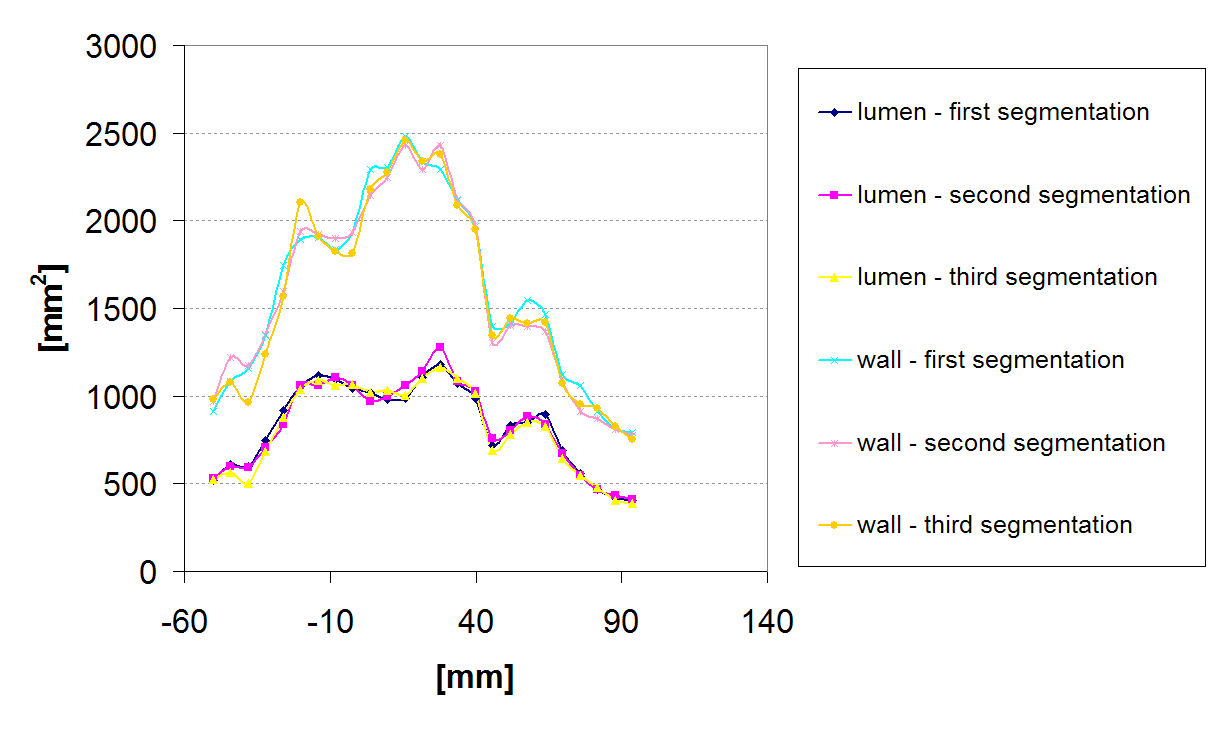
**Patient B, intra-operator dependence of the segmentation**. Comparison of lumen and wall areas from the segmentations performed at three different times (Patient B).

The results show that different types of MR images for the definition of lumen surface can result in different stress patterns along the aneurysm and different values of maximum stress. The results are of course dependent on the accuracy of image segmentation: sets A2 and B2 images were segmented automatically using the same threshold, so that the resultant contours c_LM _can be regarded as repeatable. The segmentation of sets A1 and B1 (HASTE images) was performed manually and this could be subject to operator errors. The reproducibility of intra-operator segmentation was assessed for patient B by comparing the segmented contours performed by the same operator at three different times for both lumen and wall in terms of internal area. Figure 12 shows the profile of the segmented areas for the lumen and the wall contours (c_LH _and c_W _respectively). The curves match well, with a maximum mean error of 4.6% between curves obtained from two different attempts. Since this error is lower than that allowed by the criterion adopted for the grid independence test and furthermore it is lower than the difference in maximum stress between the H and HM model for both patients, it can be assumed that the segmentation uncertainty would not affect the final results. A similar result can be expected from the other patient, since the A1 and B1 images were acquired with the same protocol and specifications.

## Discussion

The predicted stress patterns in aneurysm models are highly dependent on the reconstructed model geometry and in particular on the detailed shape of the lumen and outer wall. The segmentation of lumen contour is well established; however, the segmentation of the aortic wall and its components remains a problem to date for patient-specific model creation. Several attempts have been made to obtain wall thickness data from in vivo images, but few studies have focused on aneurysms, especially aneurysms with intraluminal thrombus. In this work, a novel method for the creation of an aneurysm model has been developed and applied to two subjects. The novelty of the method is the combination of two sets of MR images: the first set (HASTE images) allows segmentation of the arterial wall and thrombus but suffers from a low in-plane and vertical resolution so that the final result is a very coarse model of the aneurysm (see figure 2b). The second set (MR Angio sequence, acquired with subtraction technique) provides excellent details of the lumen of the aneurysm (see figure 2c) with high in-plane and vertical resolutions (see table 1 for detail); however, the use of contrast agent for the acquisition of these images makes only the lumen and other vascularised tissues visible, and the segmentation of wall and thrombus is not possible. Combining information from the two sets of images offers the opportunity to construct a model with the best resolution for both aortic wall and lumen. The procedure for incorporating the two sets of contours requires co-registration of the contours via smoothing and realignment of the centrelines of the lumen contours. Since the boundary between the aortic wall and the thrombus was not clearly visible everywhere in the image sets A1 and B1, the wall thickness has been measured where visible and then averaged for each patient. The aneurysm wall has been created as a layer with constant thickness with the segmented outer wall as external boundary. Because of the different levels of detail of the lumen segmented from the HASTE and MR Angio sequence sets, partial overlapping between the lumen surface and internal wall surface could occur in some sections especially where the circumferential distribution of the thrombus was non-uniform. Local readjustment of the contours was necessary and this led to local differences between the H and HM wall domains; however, for both patients the H and HM models gave the maximum stress at the same location and the thrombus thickness in that area was similar. The sensitivity of the predicted stress to the function parameters a, b, c used for the contour readjustment was assessed on one of the four models (model B_H_). The parameters for the readjustment of the thrombus contours were varied by at least 20% (*a *ranging from 60 degrees to 120 degrees, *b *ranging from 0.4 mm to 0.6 mm and *c *ranging from 0.6 mm to 1 mm) and the stress was calculated for each combination of values. The maximum difference between the stress of each combination and the initial case was 8.3%, which is lower than the maximum stress difference between the H and HM model for both patients. Furthermore, the average stress difference due to the parameter variation was 4.4% and the overall stress pattern does not change qualitatively; hence it is possible to confirm the validity of the comparison results. The proposed approach aimed at offering a better way to resolve the external wall and the aortic lumen boundary by merging the information from two sets of images, but an improved wall thickness measurement is needed.

The results of finite element stress analysis under a static loading condition showed differences in stress patterns between the two models due to differences in detailed geometrical features of the lumen surface. For both patients, the stress value of the coarse model was higher than that of the fine model created via two sets of images. The availability of a larger number of transverse slices and thinner slice thickness offers more details for the reconstruction of the lumen surface that captures the complicated realistic morphological features. The difference in lumen diameter between the H and HM model was examined and it was of the order of magnitude of the resolution of the HASTE images, so the difference in morphology can be attributed to the lower resolution of this image set, supporting the inferred higher reliability of the model created with both sets of images.

The resulting final shape for the wall model of the two aneurysms is fairly "smooth", due to the limited number of slices available for the creation of the external wall surface. It would be desirable to have a set of HASTE images with smaller inter-slice distance and larger number of slices, so that the outer wall surface could be reconstructed more realistically.

## Conclusion

This paper presents a novel approach for the reconstruction of aneurysm models using two different sets of MRI data. The methodology has been applied to two patients and noticeable differences between the coarse and finer models have been found. The differences in predicted maximum wall stress were found to be 30% for patient A and 11% for patient B, suggesting that detailed lumen surface representation plays an important role in determining wall stress values.

This approach enables accurate representation of the lumen while making use of patient-specific information about the wall of a TAA. This is an important step towards the development of a reliable tool for patient-specific assessment of the risk of aneurysm rupture.

## Competing interests

The author(s) declare that they have no competing interests.

## Authors' contributions

AB coded the segmentation program, created the CAD models, performed the simulations, analyzed the results and drafted the manuscript. NBW participated in the design and supervision of the study, and revised the manuscript. RHM coordinated the patient recruitment, MR image acquisition and revised the manuscript. XYX designed and supervised this study, coordinated the activities between the research group and the hospital providing the MRI data, revised the manuscript and gave the final approval. All the authors read and approved the manuscript.
